# Is Single-View Fluoroscopy Sufficient in Guiding Cardiac Ablation Procedures?

**DOI:** 10.1155/2010/631264

**Published:** 2010-03-24

**Authors:** Pascal Fallavollita

**Affiliations:** School of Computing, 557 Goodwin Hall, Queen's University, Kingston, ON, Canada K7L 3N6

## Abstract

The CARTO XP ablation system provides real-time data on 3D, color-coded maps of the electrical activity of the heart; however, it is expensive and can only use a dedicated costly magnetic catheter per patient intervention. The purpose of our study is to shorten the duration of the radiofrequency ablation procedure and increase its efficacy by developing an affordable prototype catheter navigation system that simulates the CARTO system. To obtain 3D geometrical data from catheter locations inside the heart chamber, we acquired only single-view images using an Integris Allura fluoroscope and estimated the depth of the mapping electrode using pattern recognition techniques. Validation was performed in ideal and clinical conditions. For phantom experiment, when using a 7-French catheter, the average recovered depth error was 2.05 ± 1.46 mm using a single image. However, when using the 8-French catheter, the average recovered depth error was 1.54 ± 1.29 mm. In clinical experimentation, the standard error of estimate for the estimated depth was about 13.1 mm and 10.1 mm, respectively, for the posterior and lateral views. In conclusion, this paper describes our achievements and shortfalls in developing an affordable fluoroscopic navigation system to guide RF catheter ablation of cardiac arrhythmias.

## 1. Introduction

**Figure 1:**
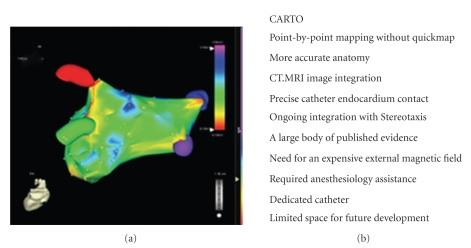
(a) Preablation colour-coded 3D maps using the CARTO electroanatomic guidance systems shown in the postero-anterior anatomic view. (b) Advantages and disadvantages of CARTO (taken from [11]).

The current incidence of sudden cardiac death (SCD) in the United States is between 200 000 and 250 000 cases per year, with a worldwide incidence of 4 to 5 million cases per year. Left ventricular dysfunction, such as ventricular tachycardia (VT) is currently the best available predictor for SCD [1]. An advanced technology called radio-frequency (RF) catheter ablation can help treat these disorders. This procedure involves inserting a catheter inside the heart and delivering RF currents through the catheter tip so as to ablate the arrhythmogenic site. Catheter ablation is now an important option to control recurrent ventricular tachycardias. The field has evolved rapidly and is continually progressing. Ablation is often a sole therapy of VT in patients without structural heart disease and is commonly combined with an implantable cardioverter-defibrillator (ICD) and/or antiarrhythmic therapy for scar-related VTs associated with structural heart disease [2]. The underlying heart disease and clinical characteristics of VT often suggest a potential mechanism and origin. Ventricular tachycardias that are due to automaticity are expected to have a focal origin, making them susceptible to ablation with discrete RF lesions [3–10]. Mapping systems that create chamber geometry and display the ablation catheter position are often helpful in ablation of VT. There are currently four main 3D mapping systems and each tool to some extent plays a part in a modern Electrophysiological (EP) laboratory to facilitate RF ablation: *CARTO* (Biosense-Webster, Diamond Bar, CA, USA), *NavX system* (St Jude Medical, St Paul, MN, USA), *RPM* (Boston Scientific, Natick, MA, USA), and *LocaLisa* (Medtronic, Minneapolis, MN, USA). Emerging technologies such as navigation and ablation with magnetic fields (*Stereotaxis*, Inc., St Louis, MO, USA) have recently been introduced [11]. All these systems including purchase of system-specific catheters are costly. Figure 1(a) shows the 3D geometry and activation color sequence displayed by the CARTO system.

Electroanatomic mapping (EAM) refers to point-by-point contact mapping combined with the ability to display the location of each mapping point in 3D. Electroanatomic mapping systems such as CARTO are used extensively in patients with VTs [12–17]. It utilizes low-level electromagnetic fields emanating from three separate coils that are measured from a location sensor embedded in the tip of the mapping catheter [18]. This allows a 3D reconstruction of the chamber of interest and colour-coded display of various electrophysiological parameters for endocardial or epicardial mapping [14, 16–20]. There are however some disadvantages with these systems. 

Although spatial accuracy in identifying the electrode-tip electromagnetically inside the chamber is at most 1-2 mm, cardiac and respiratory motions (i.e., 10–20 mm) reduce anatomic accuracy and overall 3D reconstruction of the previous. Algorithms for anatomic reconstruction differ between systems and likely have different shortfalls. Data are acquired point-by-point, such that a stable tachycardia is usually required for the definition of a complete activation sequence. Point by point mapping is a tedious process that requires considerable skill with catheter manipulation [2]. Since the CARTO nonfluoroscopic technology provides contact-based sequential acquisition of endocardial signals and reconstruction of 3D electroanatomical maps, data acquisition can be time consuming. However, this factor is operator dependent, and in experienced hands final geometry of a cardiac chamber can be obtained within 30 minutes [21]. More important, the instability of the catheter used for timing intracardiac activation and major patient movements relative to the location pad may cause the entire map to be inaccurate for subsequent use, requiring the reconstruction of a whole new map. At the present time, the major limitation for broader use of electroanatomical mapping in catheter ablation procedures is the associated cost. This is further confounded by the high cost of the mapping catheter. Figure 1(b) lists complete advantages and disadvantages for CARTO. 

The long term objective aims at developing a system similar to the functionality of the CARTO technology; albeit with the following advantages: (a) being more affordable by making use of cost effective catheters which increase the number of available types and shapes of catheters used during the cardiac ablation procedure, (b) use of common monoplane C-arm fluoroscopes compared to expensive new mapping equipment (>300 000$/system, 5000$/catheter) that need to be purchased by hospitals, and (c) that 2D/3D registration using only C-arm fluoroscopy is possible in order to superimpose a translucent image of the cardiac activation map directly over a 2D C-arm images in order to guide ablation quickly. Moreover, as biplane fluoroscopes are seldom found in hospitals and used in intervention, we propose to exploit single-view methods in hopes of reconstructing the heart chamber affected by the arrhythmia in 3D. We emphasize that our entire methodology is cost effective and this is important as the availability of expensive systems such as CARTO is still limited in developing countries [21].

The contribution of this paper is as follows: we provide a complete analysis of both electrode area and width to estimate the depth of the catheter using a novel preprocessing filtering scheme that improves results from [21]. Further, we provide a detailed report on the feasibility of using a single-view 3D reconstruction algorithm, combined with our novel preprocessing filtering scheme, to guide VT catheter ablation and present a practical implementation and experimental analysis on phantom experimentation using two types of catheters and initial results on mongrel dog data.

## 2. Methodology

### 2.1. C-Arm Fluoroscopy Geometry

#### 2.1.1. Full Perspective Projection

If we define a three dimensional point *P*_world_ = [*X* *Y* *Z* 1]^*T*^ in the world coordinate system, then its 2D projection in an image, *m* = [*u* *v* 1]^*T*^, is achieved by constructing a projection matrix:

(1) $$P_{\text{FP}}=\left[\begin{array}{ccc} kf & 0 & u_{o}\\ 0 & kf & v_{o}\\ 0 & 0 & 1 \end{array}\right]\times \left[\begin{array}{cccc} r_{11} & r_{12} & r_{13} & t_{x}\\ r_{21} & r_{22} & r_{23} & t_{y}\\ r_{31} & r_{32} & r_{33} & t_{z} \end{array}\right].$$

The intrinsic matrix of size [3 × 3], contains the pixel coordinates of the image center, also known as the principal point (*u*_*o*_, *v*_*o*_), the scaling factor *k*, which defines the number of pixels per unit distance in image coordinates, and the source-to-image distance SID, also known as the focal length, *f* of the C-arm (in meters). The extrinsic matrix of size [3 × 4] is identified by the transformation needed to align the world coordinate system to the camera coordinate system. This means that a translation vector, *t*, and a rotation matrix, *R*, need to be found in order to align the corresponding axis of the two reference frames. 

#### 2.1.2. Orthographic Projection

An orthographic camera is one that uses parallel projection to generate a two dimensional image of a three dimensional object. The image plane is perpendicular to the viewing direction. Parallel projections are an approximation to the full perspective projection; however with the advantage that parallel lines remain parallel in the projection and size does not change with respect to distance from the camera. The parallel projection matrix is given by

(2) $$P_{\text{ortho}}=\left[\begin{array}{cccc} {kr}_{11} & {kr}_{12} & kr_{13} & {kt}_{x}+u_{o}\\ {kr}_{21} & kr_{22} & kr_{23} & {kt}_{y}+v_{o}\\ 0 & 0 & 0 & 1 \end{array}\right].$$

### 2.2. Single-View 3D Reconstruction

**Figure 2:**
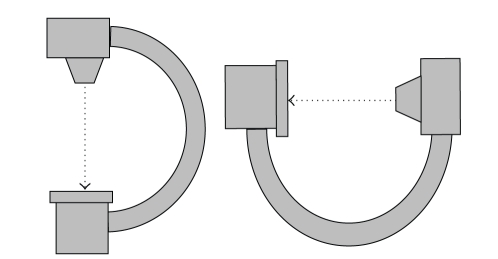
Typical C-arm fluoroscope found in hospitals. Biplane images are obtained by rotating the C-arm at two perpendicular positions, whereas monoplane images are acquires at a specific C-arm angulation.

True biplane fluoroscopy systems are rare because of their relatively high cost. Also, obtaining two projections by rotating the C-arm of a monoplane fluoroscopy system at each mapping site is cumbersome and time consuming (see Figure 2).

**Figure 3:**
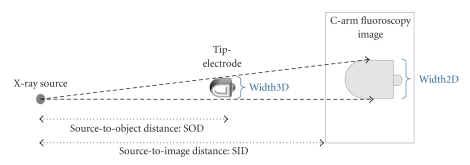
Geometric model relating the size of the electrode projection and the distance from the C-arm source.

Thus, there is a real need for computing the depth of the catheter tip from a single image. Theoretically, the depth of the tip electrode can be obtained from the width of its image since the projection of an object becomes larger as it approaches the X-ray source (see Figure 3). 

Geometric proportionality permits us to define the following equation:

(3) $$\begin{array}{cc} \frac{\text{Width}3\text{D}}{\text{width}2\text{D}} & =\frac{\text{SOD}}{\text{SID}}\to \frac{1}{\text{width}2\text{D}}\\ & =\left(\frac{1}{\text{Width}3\text{D}\times \text{SID}}\right)\text{SOD}. \end{array}$$

**Observation 1**

We deduce that the inverse of the projection width is proportional to the object depth, SOD.

**Figure 4:**
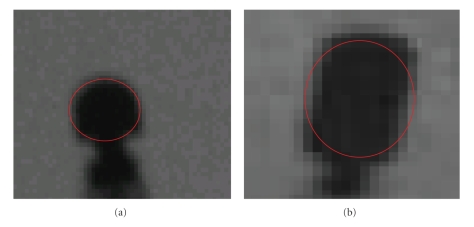
The tip-electrode of an ablation catheter approximated as a disk (ellipse).

Similarly, if we assume the tip electrode to be similar to a circle (see Figure 4), then

(4) $$\begin{array}{cc} {\text{Area}}_{\text{circle}} & =\pi r^{2}=\pi {\left(\frac{\text{width}2\text{D}}{2}\right)}^{2}\to \text{width}2\text{D}\\ & =\sqrt{\frac{4}{\pi }{\text{Area}}_{\text{circle}}}. \end{array}$$

The equation can be extended to a more general case, that of an ellipse, and is omitted here for brevity. The ellipse model fits the electrode area even if the electrode is oriented and not flat as seen in Figure 4. The elliptical model comes into play when calculating the area and width and is explained further when addressing the filtering task in the following section.

**Observation 2**

We deduce that the inverse of the square root of the area of the object is proportional to the object depth SOD.

### 2.3. Electrode Width and Area Estimation

To determine the area and width of the electrode in the 2D C-arm image, image processing techniques must be implemented. A steerable tensor voting filter to extract the tip-electrode of ablation catheters in noisy images was developed in [22]. Although results are promising as about 70% of the tip electrodes were detected successfully, determining which tip-electrode corresponds to the mapping catheter remains difficult to achieve. Thus manual selection is still necessary to collect the 2D coordinates of all tip-electrodes in the C-arm images. We propose the following approach to measure the area and width of the tip-electrode.

#### 2.3.1. Projection Approach

First we manually click on the tip-electrode from the original sized fluoroscopy image and a 60 x 60 region of interest is automatically defined around the electrode. This smaller cropped grayscale fluoroscopic image was filtered using the following sequence of filters.

A homomorphic filter is used first to denoise the fluoroscopic image [23]. The illumination component of an image is generally characterized by slow spatial variation. The reflectance component of an image tends to vary abruptly. The homomorphic filter decreases the contribution made by the low frequencies and amplifies the contribution of high frequencies. The result is simultaneous dynamic range compression and contrast enhancement. The homomorphic filter is given by

(5) $$H\left(u,v\right)=\left(\gamma_{H}-\gamma_{L}\right)\left(1-e^{-c(D^{2}(u,v)/D_{o}^{2}}\right)+\gamma_{L}$$

with *γ*_*L*_ < 1 and *γ*_*H*_ > 1. The coefficient *c* controls the sharpness of the slope at the transition between high and low frequencies, whereas *D*_*o*_ is a constant that controls the shape of the filter and *D*(*u*, *v*) is the distance in pixels from the origin of the filter.

Anisotropic systems are those that exhibit a preferential spreading direction while isotropic systems are those that have no preferences. The Perona-Malik anisotropic diffusion [24] method was implemented here in order to reduce noise and texture from the image, as well as to preserve and enhance structures. The diffusion equation is given by

(6) $$\frac{\partial I}{\partial t}=\mathrm{div}\;\left(c\left(x,y,t\right)\nabla I\right),$$

where *I* is the input image and *c*(*x*, *y*, *t*) is the diffusion coefficient that controls the degree of smoothing at each image pixel. The diffusion coefficient is a monotonically decreasing function of the image gradient magnitude. It allows for locally adaptive diffusion strengths; edges are selectively smoothed or enhanced based on the evaluation of the diffusion function. Although any monotonically decreasing continuous function of the gradient would suffice as a diffusion function, we use the following diffusion coefficient:

(7) $$c\left(x,y,t\right)=e^{-({\left(\left|\nabla I\right|/K\right)}^{2})},$$

where *K* is referred to as the *diffusion constant* or the *flow constant*. The greatest flow is produced when the image gradient magnitude is close to the value of *K*. Therefore, by choosing *K* to correspond to gradient magnitudes produced by noise, the diffusion process can be used to reduce noise in images. 

Morphological filtering was applied as a final image processing step in order to eliminate background elements around the primary coronary arteries. The structuring element consists of a pattern specified as the coordinates of a number of discrete points relative to a defined origin. Normally, Cartesian coordinates are used and so a convenient way of representing the element is as a small image on a rectangular grid. We chose a disk structuring element having a radius of a few pixels, since the contours of the tip-electrode of the mapping catheter can be modeled as a disk (see Figure 4). The structuring element will suppress the background (black) and enhance the arteries (grayscale). When a morphological operation is carried out, the origin of the structuring element is typically translated to each pixel position in the image in turn, and then the points within the translated structuring element are compared with the underlying image pixel values. 

Once these filters are applied, the final smoothed image containing the tip electrode region was rotated vertically. The values of each pixel belonging to a given column were added together, and this was repeated for every column so as to project the image along its base. This projection resulted in a bell-shaped curve. Finally, a threshold was fixed at 50% of the maximum of this curve; the two intercepts of the bell-shaped curve with the threshold were calculated by linear interpolation to estimate the width of the electrode. We then plotted the estimated width versus depth. The area of the tip-electrode resulted in calculating the number of pixels making up the bell curve and one can see that the disk structuring element used produced a filtered electrode in the shape of an ellipse as expected (see Figure 11). No matter what tip orientation may be observed in the C-arm images; the structuring element will yield a similar shaped bell curve.

### 2.4. Convex (Quick) Hull Algorithm

**Figure 5:**
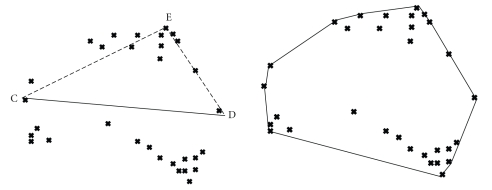
Convex Hull from a set of unorganized points.

The tip-electrode coordinates in the monoplane images are collected and used to form a 3D volume using a convex hull algorithm. The problem involves finding the smallest convex polygon containing all the points of *S*, given a set *S* of *n* points in multidimensional space [25]. The algorithm can be described briefly for 2D points as seen in Figure 5: Select the farthest points (left and right) from the data set (2D) and draw a line between them. We examine a set of points lying on the same side of the line (CD). In this set, select the point E that is located furthest away from the line CD. This point will also belong to the convex hull, since it cannot be included in a triangle. Moreover, we can remove all the points inside the triangle (CDE), and split the remaining points in two subsets: one with the points on the left of line (CE), and the other set containing points on the right of line (CE). The iterative process is repeated in these two subsets. Once the outer shell of the hull is established, we can analyze the set of data points located under the line segment (CD) in the same manner. Similar procedure can be expanded to the 3D scenario [25]. The convex hull will have an empty interior and is the method of choice in this paper. It is quick to implement and represents well the endocardial surface of cardiac chambers. Further, it was thought to be a good initial step for verifying the feasibility of our approach.

### 2.5. Evaluation

Equations (3) and (4) assume a flat shaped electrode that is projected onto the 2D image. Positioning the electrode during clinical intervention results, more often than not, in the tip-electrode being oriented either in plane or out of plane. This would result in an under or overestimating of the electrode width and areas. Two different experiments have been performed to evaluate our single view approach taking into account tip-orientation. First, in the ideal situation, we position the tip electrode so that there is no visible orientation in the acquired image, and consequently estimate the depth. Second, a mongrel dog experiment was performed and the mapping catheter was positioned with no bearing on tip orientation. We expect results to be less accurate than the ideal case, nevertheless a final analysis on the accuracy and feasibility of the method will be performed with respect to collected biplane ground truth data.

**Catheter-in-Air Experimentation**

An initial ideal experiment was performed using two different ablation catheters (7-French & 8-French) that were positioned over a radiotranslucent cardboard box and displaced at 1 cm increments away from a fluoroscopic source. The tip-electrode was cropped and preprocessed using our filter. Both area and width are calculated to determine the depth of the electrode using a single image.

**Mongrel Dog Experimentation**

Ethics board approval was obtained prior to initiating this study. A mongrel dog was anesthetized and laid on its right side on a fluoroscopy table (Integris Allura, Philips Inc.). A reference catheter and a pacing catheter were inserted into the right ventricle, close to the septal wall. The role of the reference catheter was to define an origin for our 3D coordinate system (see Figure 9(a)). This was important as motion artifacts are ever present during the experiments (heart beat, respiration, etc.), hence we deemed it appropriate to position it near a rigid landmark so that it experiences less movement due to artifacts. We recall here that although the CARTO technology determines the spatial position of the tip-electrode to within 2 mm due to expensive magnetic catheters, actual motion artifacts in the order of 10–20 mm affect the final accuracy of the 3D positions of the tip-electrodes. The role of the pacing catheter was to produce a simple electrical activation sequence so as to validate the isochronal maps. Finally, a standard 8-French RF ablation catheter was inserted from the femoral vein into the left ventricle (LV) of the dog.

During the course of the experiment, this mapping catheter was moved to 20 different sites (point-by-point) within the ventricle in order to obtain electrical and geometrical data from sufficient sites to map the activation sequence. The point-by-point technique emulates the CARTO procedure. The 20 landmarks were selected to reflect as closely as possible the entire volume of the left ventricle. Electrograms were recorded using the CardioMap software system (*Research Center at Sacré Coeur Hospital*, Montreal, Canada). Specifications for the acquisition software were: 1000 samples/second, 1000 Hz bandpass, 0.05 Hz high-pass and 450 Hz low-pass frequencies. The local activation time was measured as the difference in the times of the fastest negative deflections (dV/dt) seen in the two electrograms recorded with the reference catheter and the mapping catheter. The fluoroscopic image acquisition rate was set to 60 fps so as to minimize motion artifacts as interframe 2D images are thus closer to each other yielding a smaller displacement between the objects in consecutive images. Images were recorded during approximately 2 seconds at the end of the expiration. The monoplane fluoroscopic C-arm was rotated by 90° to acquire two biplane images for each mapping site: a left lateral view (the C-arm in a vertical position) and a posterior view (the C-arm in a horizontal position). Images were recorded with a 512 × 512 pixel resolution. We selected the diastolic frame for each mapping site so as to minimize motion blur by identifying the frame having the smallest root-mean-square difference with the preceding frame. An example of posterior/left lateral data is seen in Figure 9(b).

The results for depth estimation using the single view algorithm and novel filter proposed in this paper will be compared with the biplane ground truth: (i) first with the 3D biplane reconstruction, and second (ii) comparing it with the 2D/3D fusion maps. 

## 3. Experiments and Results

### 3.1. Catheter in Air Experiment

**Figure 6:**
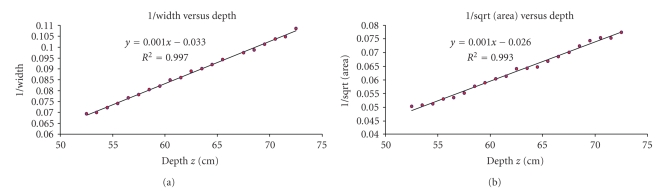
7-French catheter results. (a) The inverse of the projection electrode-tip width versus depth and (b) the inverse of the projection area versus depth using a single-view image.

Using the 7-French catheter, the depth of the catheter varied between 52.5 cm to 72.5 cm from the source. We then estimated the recovered depth using (3) by calculating the slope and intercept of the regression line in Microsoft Excel. The results show an average recovered depth error of 2.05 ± 1.46 mm when calculating the width of the tip electrode compared to 4.25 ± 2.94 mm when calculating the area. From Figure 6, almost perfect correlation is observed from the regression line.

**Figure 7:**
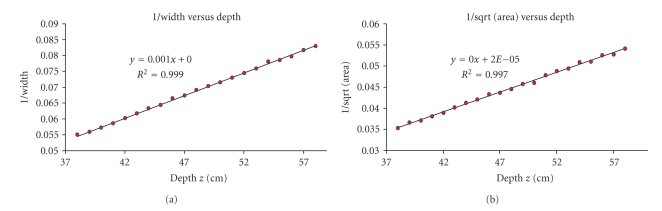
8-French catheter results. (a) The inverse of the projection electrode-tip width versus depth and (b) the inverse of the projection area versus depth using a single-view image.

Using the 8-French catheter, the depth of the catheter varied between 38 cm to 58 cm. The results obtained show an average recovered depth error of 1.54 ± 1.29 mm when calculating the width of the tip electrode compared to 2.56 ± 1.72 mm when calculating the area. From Figure 7, almost perfect correlation is once again observed from the regression line.

**Figure 8:**
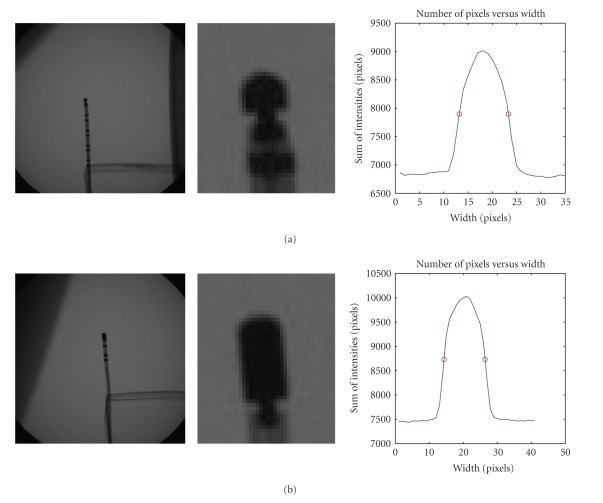
(a) 7-French Catheter with cropped tip electrode and projection of pixels along perpendicular axis. (b) 8-French Catheter with cropped tip electrode and projection of pixels along perpendicular axis.

**Figure 9:**
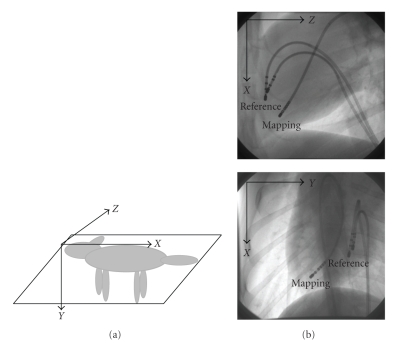
(a) Right-handed coordinate system centered on the tip of the reference catheter. (b) Posterior View: *XY* plane and Left Lateral View: *X* *Z* plane. Images represent diastolic cardiac phase.

From these results, we can conclude that the tip-electrode width yields better depth estimates using a single view. It would seem that the actual shape of the catheter affects somewhat the accuracy of depth recovery. From Figure 8, the 7-French tip electrode is more susceptible to error due to a smaller tip with a non disk shape that makes it more prone to miscalculation of width and area. The 8-French catheter provides a more accurate value, for width and area as depicted by the narrower and smoother bell shaped projection curve.

### 3.2. Mongrel Dog Experiment

We begin this section by recalling that our primary objective was to determine the feasibility of single plane 3D reconstruction to assist ventricular ablation procedures. Hence, this assessment will be made with respect to biplane data. 

At this point a decision had to be made regarding the C-arm fluoroscopy calibration technique. We opted to implement an orthographic projection scheme for two main reasons. First, we wanted to meet our third objective in that 2D/3D registration using only X-ray fluoroscopy is possible in order to superimpose a translucent image of the cardiac activation map directly over a 2D fluoroscopic X-ray image. This would serve as a reliable guide for the clinician during ablation procedures as the C-arm screens are being observed at all times during catheter navigation inside the heart chamber. Thus, the C-arm images are not fully calibrated because there is no need to transform the pixel values of the image coordinates into millimeters of a “world” coordinate system because isochronal maps are always plotted in the image coordinates for superposition with the fluoroscopic data. Second, as this is a feasibility study, the orthographic projection scheme is easy to implement and reproduce.

**Figure 10:**
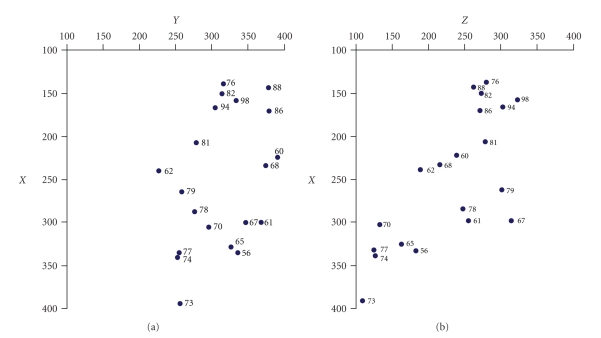
2D pixel coordinates for 20 locations visited by mapping tip-electrode in left ventricle as seen in the posterior and left lateral images. Isochronal times are tagged at the 20 sites.

The distribution of all 20 mapping sites is shown in Figure 10 in the 2D case for both posterior and lateral views. The axes are in image pixel coordinates and the associated activation times are labeled in blue.

#### 3.2.1. Clinical Error Validation

**Figure 11:**
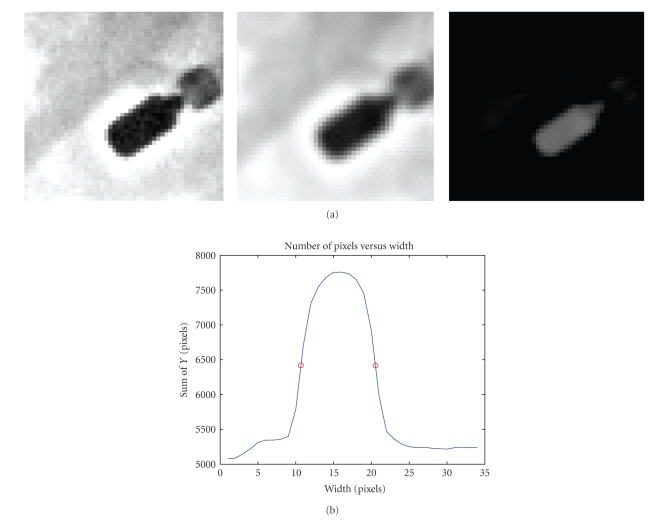
Projection approach applied to the tip electrode of an ablation catheter. (a) Homomorphic, anisotropic and morphological filter applied to tip electrode and (b) vertical projection of the electrode producing a bell shape curve used to estimate electrode width and area.

As electrode width or area is measured in pixels from the images, using the proposed filtering technique (see Figure 11) and the standard error of estimate from our results, we can estimate the depth error in millimeters as follows: (i) the width of electrode tip is approximately 2 mm in this experiment from 8-French catheter, (ii) we calculate the average width in pixels over the 20 sites visited by the mapping catheter, (iii) we divide the standard error of estimate by the average width, and (iv) we multiply this value by the 2 mm electrode width to obtain an error in millimeters. 

**Figure 12:**
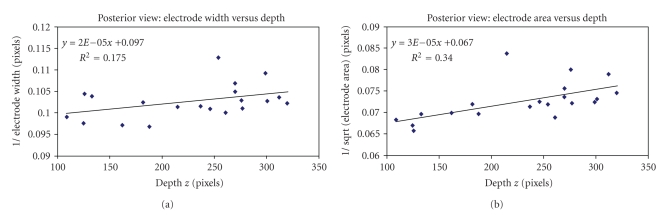
(a) Inverse of the projection width *w* versus depth *z*. (b) Inverse of the projection area A versus depth *z*. The area calculation yields statistically significant result when compared to width calculation.

Using the posterior view images, the standard error of estimate for the width calculation yielded a value of SE = 63.76 pixels. The mean width of the tip electrode was *μ*_width_ = 9.76 pixels. Since the catheter diameter (width) was 2 mm, this indicates that the standard error of estimate of the depth recovered was 63.76/9.76 × 2, which is about 13.1 mm for the posterior view. From Figure 12, the correlation coefficient was *r* = 0.42 and *r* = 0.58, respectively, using the calculated width and area of the tip-electrode from our image processing technique. 

**Figure 13:**
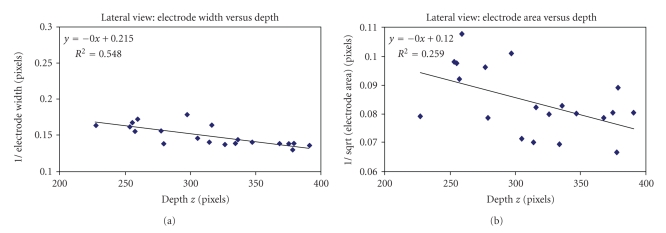
(a) Inverse of the projection width *w* versus depth *z*. (b) Inverse of the projection area A versus depth *z*. The width calculation yields statistically significant result when compared to width calculation.

Using the left lateral images, the standard error of estimate for the width calculation yielded a value of SE = 34.35 pixels. The mean width of the tip electrode was *μ*_width_ = 6.77 pixels. Since the catheter diameter (width) was 2 mm, this indicates that the standard error of estimate of the depth recovered was 34.35/6.77 × 2, which is about 10.1 mm for the left lateral view. From Figure 13, the correlation coefficient was *r* = 0.74 and *r* = 0.51, respectively, using the calculated width and area of the tip-electrode from our image processing technique. Visual assessment from both data showed better contrast for the left lateral view, probably due to the oblong section of the mongrel dog torso, which might explain the better results for the depth estimation.

To obtain 3D geometrical data about catheter locations from 2D images, we first used a biplane approach, then a single-view approach. For the biplane analysis, we had to sequentially rotate the monoplane C-arm fluoroscope in order to acquire two perpendicular images. Once this was done, we calculated the 2D coordinates of the mapping catheter in each of the posterior and left lateral view images by manually clicking on the tip-electrode. Then, we assumed a true biplane setup and kept the *X* coordinate the same for both images in order to obtain the 3D *XYZ* positions of the catheters.

**Figure 14:**
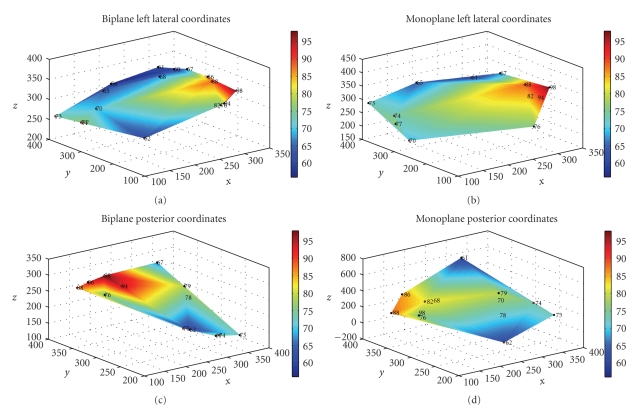
(a), (c) Biplane ground-truth convex hull for left lateral view and posterior view respectively. (b), (d) Single-view 3D estimations of the left ventricle using the convex hull algorithm. We note that the posterior volume estimation is less precise compared to ground truth.

**Figure 15:**
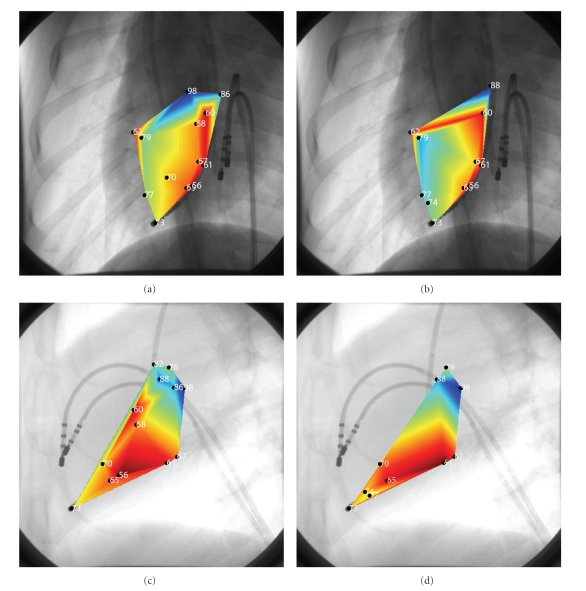
2D/3D Registration sample images. (a, (c) Ground truth isochronal maps superimposed directly on the first dataset in the posterior view and left lateral view respectively. (b), (d) Single-view isochronal maps stemming from the convex hull demonstrate that isochronal times disappear in both the posterior view and left lateral views due to depth estimates of about 10 mm. However, fastest times are still near pacing catheter using the monoplane depth estimation approach.

For the single-view analysis, we proposed the projection method with concurrent filtering. The results showed depth estimation errors of roughly 1 cm using only a single image. This affected the final geometry of the convex hull; however, surprisingly the isochronal maps still depicted fastest times and coordinates (red) close to the pacing catheter when compared to the ground-truth isochronal maps obtained from the biplane analysis (see Figures 14 and 15). 

This raises an interesting question as to the validity of our monoplane analysis when compared to the CARTO technology. While the spatial accuracy of the tip electrodes for these systems is below 2 mm; nevertheless, motion artifacts between 10–20 mm still need to be accounted for during arrhythmias representing a potential large shift in the actual position of the tip-electrode. This implies inaccurate 3D anatomical reconstruction using CARTO. By using inexpensive catheters and only a monoplane C-arm fluoroscope, we extract the information directly from the C-arm images that already contain motion artifacts (i.e., respiration and heart movement) and obtain final depth estimates in the order of 10–20 mm similar to CARTO. Clearly from Figures 14 and 15, using the left lateral view to guide ablation of the ventricle seems at first sight achievable as the convex hull geometry looks roughly the same and the 2D isochronal maps depict with sufficient clarity the activation of the ventricle. To correct for motion artifacts due to the beating of the heart as well as the patient's respiration, we acquired the fluoroscopic images at the end of expiration and we used a reference catheter that was left in the right ventricle while we mapped the left ventricle with a separate catheter.

Two dimensional isochronal activation maps were superimposed over the corresponding fluoroscopic images. This innovation is not found in either CARTO or other technologies outlined in the Introduction. These isochronal maps accurately depicted the progression of the electrical activation away from the pacing catheter using the biplane approach but still depicted a reasonable colormap with the single-view method. Image contrast affects the accuracy of the depth estimates: visual analysis revealed that the left lateral images always had a better contrast than the posterior images because of the oblong section of the mongrel dog torso, and these left lateral images provided better depth estimates. Tip orientation affected the accuracy of the width and area calculations and degraded the depth estimates in clinic as expected. To alleviate the affect of noise on depth estimates, we implemented a 4-step filter when obtaining the bell shaped curve from the tip-electrode. Observed results were better than those presented in [21]. 

It appears that by using standard equipment such as inexpensive catheters and our prototype data acquisition system, there is a strong possibility to simulate at a much lower cost the function of the CARTO system presently on the market. We recall that our entire methodology is cost effective and this is important as the availability of expensive systems such as CARTO is still limited in developing countries [21]. Our approach however remains to be tested on human patients first. Also, data acquisition required approximately 30–40 minutes with the biplane approach, hence acquisition time could be reduced in half using our single-view approach. We conclude by stating that the 10 mm depth error seems to be a worst case error in the mongrel dog experiment as the tip-electrodes do not remain in a flat position inside the ventricle but are often slightly oriented. The presented filtering technique substantially reduces neighboring objects and noise around the tip electrode to yield excellent depth estimates in air and adequate depth errors in the mongrel dog experiment when using only a single image.

## 4. Conclusion

This paper describes our achievements and shortfalls in the development of an affordable fluoroscopic navigation system to guide RF catheter ablation of cardiac arrhythmias. Early experience in the air experiment showed that there is a direct relationship between tip-electrode width/area and catheter depth using a single image as expected. Several conclusions can be drawn from the present work: (i) only C-arm images are used to detect 3D catheter positions with no added cost from expensive 3D mapping technologies and catheters, (ii) a new filtering scheme to suppress artifacts surrounding the mapping catheter tip-electrode improved results from previous work when estimating electrode depth, (iii) both the width and area of the tip electrode are used to approximate the depth position of the tip-electrode of a mapping catheter to 1 cm accuracy which also accounts for tip-orientations not being flat in the mongrel dog experiment, (iv) the left lateral view image acquisition protocol provided the best 3D left ventricle reconstructions and depth estimates using a single view approach since the images were of better quality, and (v) we superimposed activation maps with 1 cm depth errors directly on the left lateral images and visually determined that the maps were similar to the ground truth (i.e., biplane constructed isochronal maps). Further quantitative and robust analysis on patient data is needed to corroborate these findings for feasibility and potential use of our proposed navigation findings.
